# Preoperative localization in primary hyperparathyroidism: a stepwise approach integrating PTH-FNA washout and second-line ^18^F-choline PET/CT

**DOI:** 10.3389/fendo.2026.1926105

**Published:** 2026-08-11

**Authors:** Antonino Russo, Marco Erini, Davide Donner, Fabio Magnani, Silvia Fasanella, Pierluigi Amadori, Carlo Cosimo Quattrocchi, Vito Racanelli

**Affiliations:** 1 Internal Medicine Division, “Santa Chiara Hospital”, Azienda Sanitaria Universitaria Integrata del Trentino (ASUIT), Trento, Italy; 2 Department of Radiological Sciences and Medical Imaging, Unit of Nuclear Medicine - Trento, Azienda Sanitaria Universitaria Integrata del Trentino (ASUIT), Trento, Italy; 3 Department of Integrated Activities (DAI) of Pathology and Laboratory Medicine, Multizonal Unit of Clinical Pathology Laboratory, Azienda Sanitaria Universitaria Integrata del Trentino (ASUIT), Trento, Italy; 4 Unit of Primary Care, Azienda Sanitaria Universitaria Integrata del Trentino (ASUIT), Trento, Italy; 5 Department of Radiological Sciences and Medical Imaging, Unit of Radiology - Rovereto, Azienda Sanitaria Universitaria Integrata del Trentino (ASUIT), Trento, Italy; 6 Center for Medical Sciences, University of Trento, Trento, Italy

**Keywords:** ^18^F-choline PET/CT, MIBI-Scan, minimally invasive parathyroidectomy, parathyroid adenoma, primary hyperparathyroidism, PTH-FNA washout, ultrasonography

## Abstract

**Objective:**

Accurate preoperative localization of parathyroid adenomas is essential for the success of minimally invasive parathyroidectomy. This study evaluated the detection rate of parathyroid hormone assay on fine-needle aspiration washout and in conventional and advanced imaging modalities modeling a hypothetical stepwise diagnostic approach for primary hyperparathyroidism.

**Methods:**

We conducted a retrospective single-center study including 32 patients with primary hyperparathyroidism who underwent minimally invasive parathyroidectomy and preoperative parathyroid hormone assay on fine-needle aspiration washout. We used the PTH-FNA washout to serum PTH ratio as the cutoff: a value greater than 1 was considered indicative of positive localization, while a value equal to or less than 1 was considered negative. Preoperative localization procedures included ultrasonography, MIBI-Scan, and ^18^F-choline PET/CT. Results were compared with postoperative histology and biochemical outcome.

**Results:**

Parathyroid hormone assay on fine-needle aspiration washout was positive in 20 of 32 patients (62.5%, 95% CI 45.3–77.1%). Among washout-positive patients, 7 of 20 (35.0%) underwent surgery based on ultrasonography and washout findings alone, with histological confirmation of parathyroid adenoma in all cases. In the washout-positive subgroup in whom MIBI-Scan was also performed, scintigraphy was diagnostic in 7 of 13 cases (53.8%). In washout-negative patients, scintigraphy localized the lesion in 6 of 12 cases (50.0%). In patients with non-localizing washout and scintigraphy findings, ^18^F-choline PET/CT identified the lesion in all evaluated cases. In this retrospective series, a stepwise strategy based on ultrasonography combined with needle-aspiration washout as the first-line localization, followed by ^18^F-choline PET/CT in washout-negative cases, could have potentially avoided MIBI-scans in 25 out of 32 patients (78.1%; 95% CI 61.2–89.0%).

**Conclusion:**

This retrospective, single-center, *post-hoc* reconstructed cohort study might suggest a stepwise approach for preoperative localization of parathyroid adenoma based on parathyroid hormone assay on fine-needle aspiration washout combined with ultrasonography as a useful first-line diagnostic technique and reserving ^18^F-choline PET/CT for non-localizing cases. It may reduce the use of additional imaging and radiation exposure. Larger prospective studies are needed to confirm these findings.

## Introduction

Primary hyperparathyroidism (PHPT) is an endocrine disorder caused by solitary or multiple hyperfunctioning parathyroid lesions (hyperplasia, adenomas, or, rarely, carcinomas). It has an estimated prevalence of 176 cases in women and 77 cases in men per 100,000 people per year (1995-2010) (1). In Western Europe, as in the United States, the incidence of unspecified hyperparathyroidism increased steadily to 40.3 per 100,000 woman‐years and 13.7 per 100,000 man‐years (2). PHPT has many clinical implications, such as hypercalcemia, decreased bone mineral density, and kidney stones (3). Typically, it is diagnosed through laboratory testing, revealing elevated PTH levels and increased serum calcium concentrations (4). Accurate preoperative localization of the diseased gland is the key challenge for successful surgical treatment. Currently, the preferred surgical method is minimally invasive parathyroidectomy (MIP), primarily due to the predominant etiology of a single adenoma. The success of MIP depends upon the precision of the imaging modalities used for parathyroid adenoma (PA) localization (5).

At present, the diagnostic standard for PHPT includes the use of neck ultrasonography (US) and ^99m^Tc-MIBI scintigraphy (MIBI-Scan) (5). US is a widely available, non-invasive, and inexpensive localizing procedure, but its success is highly dependent on the experience of the ultrasonographer (6). MIBI-Scan is the most frequently used nuclear medicine imaging modality for the accurate localization of PA due to its good sensitivity and specificity (7). In equivocal or inconclusive cases, positron emission tomography/computed tomography with ^18^F-choline (^18^F-choline PET/CT) offers higher sensitivity than conventional US and MIBI-Scan; a meta-analysis conducted by Treglia et al. reported a pooled sensitivity and predictive positive value (PPV) of 92% (95% CI: 88–96%) and 92% (95% CI: 89–95%) on a per-lesion analysis, respectively (8). However, its broader use is limited by high costs and low availability (9). A further effective method, which has gained traction in the last decade, is the so-called 4D-CT, which consists of the serial evaluation of subsequent contrast medium phases to identify the typical parathyroid HPT pattern (10). The diagnostic strengths of this method are linked to its very high spatial resolution and the capability to define anatomical features as well as to obtain a clear visual separation, even between smaller structures. In this context, CT can help identify a hyperfunctioning HPT even in close proximity to a large multinodular goiter, defining the posterior profile of the thyroid capsule or identifying a plane of cleavage between structures (11). The last two methods mentioned above have shown a great detection rate and sensitivity; however, they are to this day less widespread than the first-line one due to high resource and cost requirements, radiation exposure, and limited accessibility (7, 9, 10).

A promising method to confirm parathyroid adenoma localization involves evaluating the PTH concentration in the washout from an ultrasound-guided fine-needle aspiration biopsy (PTH-FNA washout). Although PTH-FNA washout is a minimally invasive procedure that carries potential localized side effects (bleeding, infections, or recurrent nerve paresis), these risks remain exceedingly rare when performed by experienced operators (12). Furthermore, this procedure demands high operator expertise to ensure precise needle placement within the suspicious lesion, which is essential to minimize the risk of false-negative results. A few studies have investigated and reported the benefits of using this US-guided procedure in a clinical setting; however, it remains unclear whether PTH-FNA washout should be an adjunct or an alternative to conventional imaging modalities like the MIBI-Scan (13). For PTH-FNA washout, a sensitivity of 70–100% and a specificity of 75–100% have been published, rendering it more reliable than cytology alone (14). Current international consensus guidelines from the Fifth International Workshop on the Evaluation and Management of Primary Hyperparathyroidism, along with the accompanying statement on surgical aspects of the disease, recommend preoperative imaging localization prior to minimally invasive parathyroidectomy, without endorsing a single mandatory modality (15, 16). At the national level, the 2024 position statement of the United Italian Society of Endocrine Surgery (SIUEC) similarly identifies diagnostic localization studies — including cervical ultrasonography, MIBI-Scan, and ^18^F-choline PET/CT — as a central component of the preoperative work-up, while leaving the choice and sequence of modalities to institutional expertise and resource availability (17). Within this framework, PTH-FNA washout has not yet been formally incorporated into consensus algorithms, representing the knowledge gap this study seeks to address.

The recent real-world evidence provided by Fries et al. serves as the ideal springboard for our study, as it underscores the clinical necessity of transitioning from traditional, often inconclusive first-line imaging to a structured, stepwise diagnostic algorithm. Specifically, they evaluated the real-world diagnostic performance of various preoperative modalities—including ultrasound, MIBI-Scan, and ^18^F-choline PET/CT—by comparing their individual and combined sensitivity in accurately localizing hyperfunctioning parathyroid glands. Crucially, Fries et al. demonstrated that conventional first-line imaging (ultrasound and MIBI-Scan) yields a conclusive localization in only about half of the patients, while simultaneously proving that ^18^F-choline PET/CT achieves an outstanding 100% sensitivity in resolving challenging and inconclusive cases (18).

The aim of this study was to assess the detection rate of PTH-FNA washout in the preoperative work-up of primary hyperparathyroidism and to explore whether it may be used within a stepwise localization strategy to optimize the use of conventional nuclear imaging techniques.

## Materials and methods

### Study design

We performed a retrospective analysis of 211 patients diagnosed with PHPT and evaluated at our outpatient clinic from January 2023 to June 2025. Of these, 122 patients who underwent parathyroidectomy were selected. Finally, we analyzed the subset of 32 patients who underwent PTH-FNA washout during their preoperative diagnostic work-up. Compared with the 90 operated patients who did not undergo washout, the study cohort was, by definition, characterized by a 100% rate of ultrasound-detectable lesions, versus 80.9% in the non-washout group (Fisher’s exact test, p = 0.006). Age, sex distribution, preoperative PTH, and calcium levels were otherwise comparable between groups, suggesting that selection for washout was primarily driven by lesion visibility on ultrasound rather than by broader clinical or biochemical differences. PTH-FNA washout was performed at the discretion of the treating clinicians in patients with ultrasound-detectable lesions considered suitable for needle sampling.

**Figure 1:**
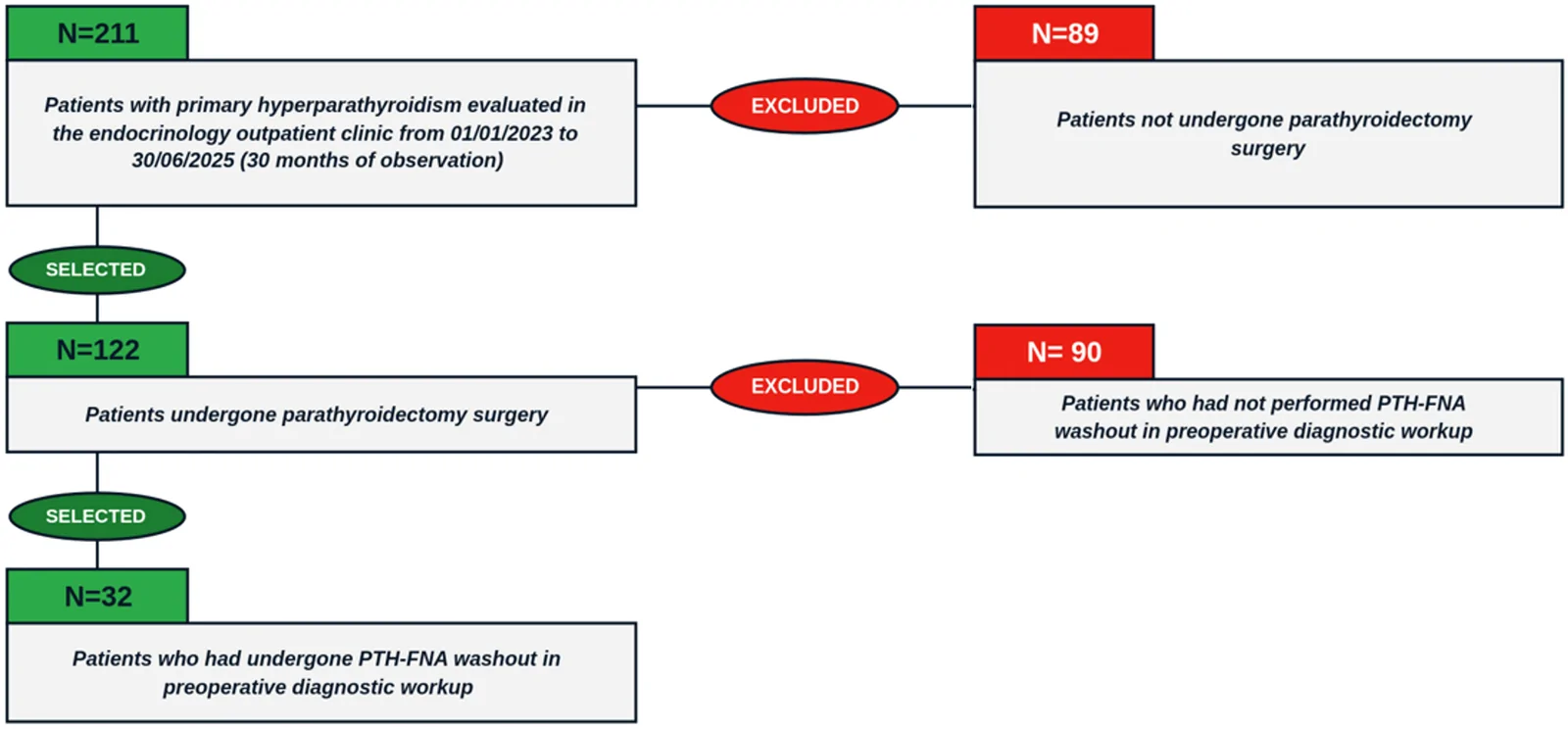
Patient selection flowchart. Of 211 patients with primary hyperparathyroidism (PHPT) evaluated at our outpatient clinic between 01/01/2023 and 30/06/2025 (30 months of observation), 122 underwent parathyroidectomy; of these, 32 underwent PTH-FNA washout (parathyroid hormone assay on ultrasound-guided fine-needle aspiration washout) as part of their preoperative diagnostic work-up and constitute the study cohort analyzed in this manuscript. N, number of patients.

The primary outcome was successful preoperative lesion localization, as confirmed by surgical findings, histopathology, and postoperative biochemical cure, defined as normalization of postoperative serum calcium levels during follow-up. All patients had single-gland disease and did not experience a recurrence of hypercalcemia after surgery. Because all 32 patients in the study cohort had surgically and histologically confirmed single-gland disease, no true negative cases were available by design; consequently, this study can estimate only detection rate (a form of sensitivity) but cannot estimate specificity or negative predictive value (NPV), and these terms are not used to describe the study’s own results anywhere in this manuscript. The patient selection flowchart is shown in **Figure 1**.

### Data collection

The following data were collected: demographic information, US imaging findings, preoperative PTH-FNA washout levels, MIBI-Scan results, ^18^F-choline PET/CT results, and pre- and postoperative serum parameters (creatinine, serum PTH, total calcium, ionized calcium, phosphate, 25-OH vitamin D). Neck ultrasonography reports were reviewed for the diameter and localization of the suspected parathyroid lesions. Histopathology data were also recorded.

### Definition of a diagnostic/positive MIBI-Scan and ^18^F-choline PET/CT

A MIBI-Scan or ^18^F-choline PET/CT scan was classified as diagnostic/positive when the original clinical report described a focal area of abnormal radiotracer uptake compatible with a hyperfunctioning parathyroid gland concordant with the surgical localized site. Scans were interpreted by the reporting nuclear medicine physician as part of routine clinical care and were not re-read in a blinded fashion for this study; readers were therefore not blinded to the results of ultrasonography or PTH-FNA washout, which is addressed as a limitation below.

### PTH-FNA washout procedure

FNA of the parathyroid gland was performed under ultrasound guidance using a 7.5-MHz linear transducer (LOGIQ S7 Expert, General Electric). Before the FNA, the skin was cleansed with a povidone-iodine solution. A standard 23-gauge needle was inserted using a freehand technique. The needle was moved several times within the mass (specifically for parathyroid glands appearing hyperplastic on ultrasound), and material from the target lesion was obtained through capillary action without applying suction. The needle was then rinsed in 1 cc of isotonic saline solution, placed inside a standard plastic blood test tube, and submitted to the laboratory for the PTH assay. To ensure precise intra-lesional needle placement and minimize the risk of false-negative outcomes, all PTH-FNA procedures were performed under continuous, real-time ultrasound guidance by experienced operators, confirming that the needle tip was strictly positioned within the target lesion throughout the aspiration process. No patient developed complications after the procedure. Currently, there are no established reference ranges for PTH-FNA washout defined at our institution or in the literature. We used the PTH-FNA washout to serum PTH ratio as the cutoff: a value greater than 1 was considered indicative of positive localization, while a value equal to or less than 1 was considered negative. This threshold was chosen pragmatically, in the absence of a validated institutional or literature-based reference range, on the biological rationale that any washout PTH concentration exceeding the patient’s own circulating serum PTH level indicates that the sampled tissue is actively contributing PTH above systemic baseline, consistent with parathyroid origin. We deliberately favored this permissive threshold over the markedly stricter criteria used by other groups (e.g., washout >400 pmol/L and ≥3-fold serum PTH in Moise et al. (19), or values above the normal serum range in Maser et al. (20)) because, within our diagnostic algorithm, PTH-FNA washout was intended to function as a first-line screening step rather than a stand-alone confirmatory test: a positive result prompted further evaluation MIBI-Scan, or ^18^F -choline PET/CT, rather than an immediate, unconfirmed surgical decision in the majority of cases (13/20, 65%, proceeded to MIBI-Scan despite a positive washout). All PTH concentrations in this manuscript (serum and washout) are reported in pg/mL, consistent with our laboratory’s intact-PTH assay; where relevant for comparison with studies reporting PTH in SI (International System) units, values were converted using the standard factor for intact (1-84) PTH (molecular weight 9,424 Da): pg/mL × 0.1061 = pmol/L (equivalently, 1 pmol/L ≈ 9.43 pg/mL).

### Statistical analysis

Continuous variables were tested for normality using the Shapiro-Wilk test; given the small sample size (n = 32, with subgroups as small as n = 12) and the presence of non-normal distributions for several variables, all continuous variables were summarized descriptively as median and interquartile range (IQR) and compared using the non-parametric Mann-Whitney U test for consistency, regardless of the individual normality test outcome, to avoid selectively applying parametric methods only to variables that passed normality testing. Categorical variables are presented as counts and percentages. Between-group comparisons for categorical variables were performed using Fisher’s exact test, preferred over the chi-square test whenever any expected cell count was below 5 (frequent given the sample size of this study), and the chi-square test otherwise. A two-sided p value < 0.05 was considered statistically significant. Statistical analyses were performed using JASP version 0.19.3.

## Results

### Demographics and disease characteristics

A total of 32 patients were included in the study. Demographic and clinical characteristics are summarized in **Table 1**. The median [IQR] age was 66.1 [61.3-71.6] years, and 26 (81.3%) were female. Histopathological examination indicated a single adenoma in 32 (100%) patients. MIBI-Scan results were available for 25 (78.1%) patients, and ^18^F-choline PET/CT for 13 (40.6%). The median preoperative serum total calcium, ionized calcium, inorganic phosphate, 25(OH) vitamin D, and PTH levels were 10.85 mg/dL [10.6-11.22], 5.34 mg/dL [5.0-5.74], 2.7 mg/dL [2.38-2.9], 26.5 ng/mL [19.5-32.25], and 134 pg/mL [95.5-170.5], respectively. The median postoperative serum total calcium was 9.4 mg/dL [9.1-9.7] and the median postoperative serum PTH was 61.0 pg/mL [47.0-91.0].

**Table 1:** Patients’ demographics, clinical characteristics and biochemical parameters.

Parameter	n=32
***Age, years, Median (IQR)***	66.1 (61.3-71.6)
Sex n(%)	
*Female*	26 (81.3%)
*Male*	6 (18.7%)
Lesion side, n(%)	
*Right Side*	16 (50.0%)
*Left Side*	16 (50.0%)
PTH Washout/serum PTH Ratio, n(%)	
*> 1*	20 (62.5%)
*<= 1*	12 (37.5%)
MIBI-Scan n(%)	
*Available*	25 (78.1%)
*Not Available*	7 (21.9%)
^18^F-Choline PET/CT N(%)	
*Available*	13 (40.6%)
*Not Available*	19 (59.4%)
***Size of suspected parathyroid in US (major diameter in mm) Median (IQR)***	11.5 (9.75-16.0)
***Preoperative Serum Total Calcium (mg/dl) Median (IQR)***	10.85 (10.6-11.22)
***Preoperative Serum Ionized Calcium (mg/dl) Median (IQR)***	5.34 (5.0-5.74)
***Preoperative Serum Phosphate (mg/dl) Median (IQR)***	2.7 (2.38-2.9)
***Preoperative Serum 25 OH Vitamin D (ng/ml) Median (IQR)***	26.5 (19.5-32.25)
***Preoperative Serum Creatinine (mg/dl) Median (IQR)***	0.78 (0.68-1.02)
***Preoperative Serum PTH (pg/ml) Median (IQR)***	134 (95.5-170.5)
***Postoperative serum Calcium (mg/dl) Median (IQR)***	9.4 (9.1-9.7)
***Postoperative serum PTH (pg/ml) Median (IQR)***	61 (47-91)
***PTH FNA Washout Level (pg/ml) Median (IQR)***	519.5 (27.75-1425.5)

n, number; IQR, interquartile range.

### Diagnostic work-up

**Figure 2:**
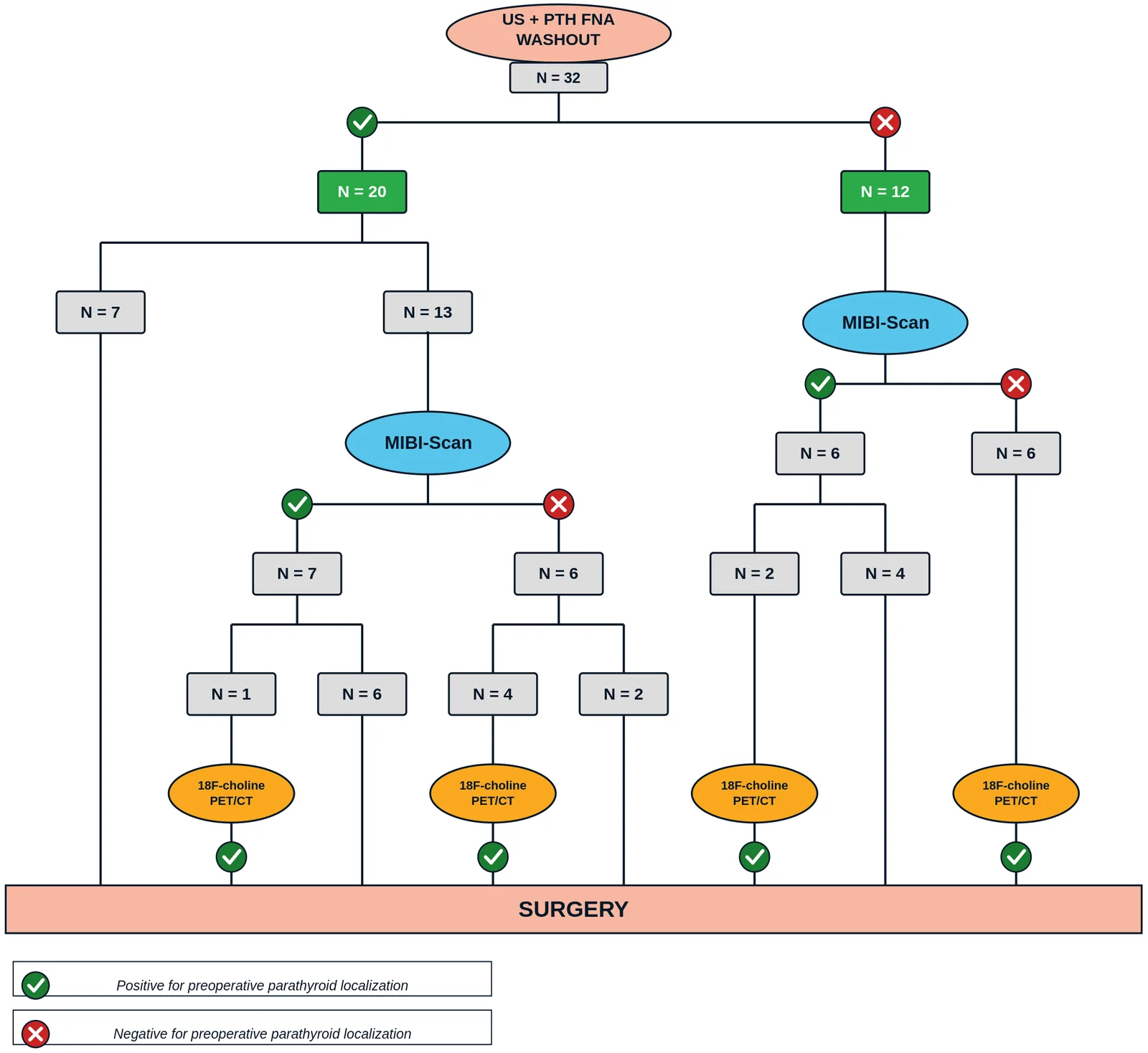
Diagnostic pathway and localization outcomes. Flow of the 32 study patients through PTH-FNA washout (parathyroid hormone assay on fine-needle aspiration washout), subsequent MIBI-Scan and ^18^F-choline PET/CT where performed, and final surgical outcome. The green check mark (✓) indicates a positive result and the red cross (✗) indicates a negative result for the specific test performed at that node. ‘Positive’ and ‘negative’ refer to the original clinical interpretation of that test at the time it was performed (presence or absence of focal findings compatible with a hyperfunctioning parathyroid gland concordant with the surgical localized site lesion). The reference standard used to adjudicate true localization accuracy was surgical and histopathological confirmation of the affected parathyroid gland. All pathways converge on surgery (bottom bar). N, number of patients.

We analyzed the preoperative diagnostic work-up of all patients starting with the PTH-FNA washout (**Figure 2**). In 20/32 patients (62.5%, 95% CI 45.3–77.1%), the PTH-FNA washout was positive, while in 12/32 patients (37.5%) it was negative.

### Positive PTH-FNA washout group

Within the positive group, 7/20 (35%, 95% CI 18.1-56.7%) were referred for surgery without further diagnostic tests; 13/20 (65%, 95% CI 43.3-81.9%) underwent MIBI-Scan, which was diagnostic in only 7/13 (53.8%, 95% CI 29.1-76.8%) cases; among them, one patient also underwent ^18^F-choline PET/CT scan, which confirmed the location. Of the remaining 6 patients who tested positive on PTH-FNA washout but negative on MIBI-Scan, 4/6 (66.7%) underwent ^18^F-choline PET/CT, which was localizing in all cases, whereas 2/6 (33.3%) proceeded directly to surgery without ^18^F-choline PET/CT confirmation.

### Negative PTH-FNA washout group

Within the negative group, all 12 patients underwent a MIBI-Scan, which was positive in only 6/12 (50%). The 6/12 (50%) who tested negative on both the PTH-FNA washout and MIBI-Scan underwent ^18^F-choline PET/CT, which was positive in 100% of cases. Additionally, ^18^F-choline PET/CT was performed for confirmation in 2/12 (16.6%) patients who were negative on the washout but positive on the MIBI-Scan, confirming the localization in both instances.

### Patient’s characteristics according to PTH-FNA washout result

A comparison of the demographic, clinical, and laboratory characteristics between the positive and negative washout groups revealed no significant differences, except for the PTH-FNA washout levels themselves (p < 0.001) (**Table 2**).

**Table 2:** Comparison of patients’ demographics, clinical characteristics and biochemical parameters between Positive PTH-FNA washout Group and Negative PTH-FNA washout.

Parameter	Positive PTH-FNA Washout Group (N = 20)	Negative PTH-FNA Washout Group (N = 12)	p
***Age (years), median (IQR)***	*66.6 (61.3-72.2)*	*64.3 (61.3-68.7)*	*0.683*
***Female Sex (N, %)***	*17 (85%)*	*9 (75%)*	*0.815*
***Lesion side (N, %)***			
***Right Side***	*9 (45%)*	*7 (58.4%)*	*0.206*
***Left Side***	*11 (55%)*	*5 (41.6%)*	
***Size of suspected parathyroid in US (mm) median (IQR)***	*12.0 (10.0-14.5)*	*10.5 (9.0-16.25)*	*0.412*
***Preoperative Serum Total Calcium (mg/dl) median (IQR)***	*10.85 (10.6-11.15)*	*10.75 (10.48-11.22)*	*0.710*
***Preoperative Serum Ionized Calcium (mg/dl) median (IQR)***	*5.25 (5.0-5.63)*	*5.46 (5.15-5.82)*	*0.370*
***Preoperative Serum Phosphate (mg/dl) median (IQR)***	*2.75 (2.48-2.98)*	*2.55 (2.28-2.72)*	*0.148*
***Preoperative Serum 25 OH Vitamin D (ng/ml) median (IQR)***	*25.5 (20.0-32.25)*	*28.0 (17.75-33.75)*	*0.815*
***Preoperative Serum Creatinine (mg/dl) median (IQR)***	*0.8 (0.66-1.1)*	*0.78 (0.69-0.94)*	*0.755*
***Preoperative Serum PTH (pg/ml) median (IQR)***	*146 (95.5-207.75)*	*128.5 (95.0-150.75)*	*0.284*
***Postoperative Serum Calcium (mg/dl) median (IQR)***	*9.3 (9.1-9.7)*	*9.6 (9.15-9.98)*	*0.439*
***Postoperative Serum PTH (pg/ml) median (IQR)***	*61.0 (47.0-113.5)*	*56.5 (48.0-70.25)*	*0.57*

n, number; IQR, interquartile range. p value: categorical variables compared using Fisher’s exact test; continuous variables compared using Mann-Whitney U test.

### Localization rate

An overall analysis of the data showed that the PTH-FNA washout localized the adenoma in 20/32 patients (62.5%, 95% CI 45.3–77.1%); the MIBI-Scan confirmed the localization in 13/25 (52%, 95% CI 33.5–70%). Among the 25 patients who underwent both PTH-FNA washout and MIBI-Scan, a paired comparison (McNemar’s exact test) showed no statistically significant difference between the two modalities (p = 1.00; 6 patients PTH-FNA washout-positive/MIBI-Scan-negative and 6 PTH-FNA washout-negative/MIBI-Scan-positive), indicating that, within this small paired subsample, neither test can be considered superior to the other.

**Figure 3:**
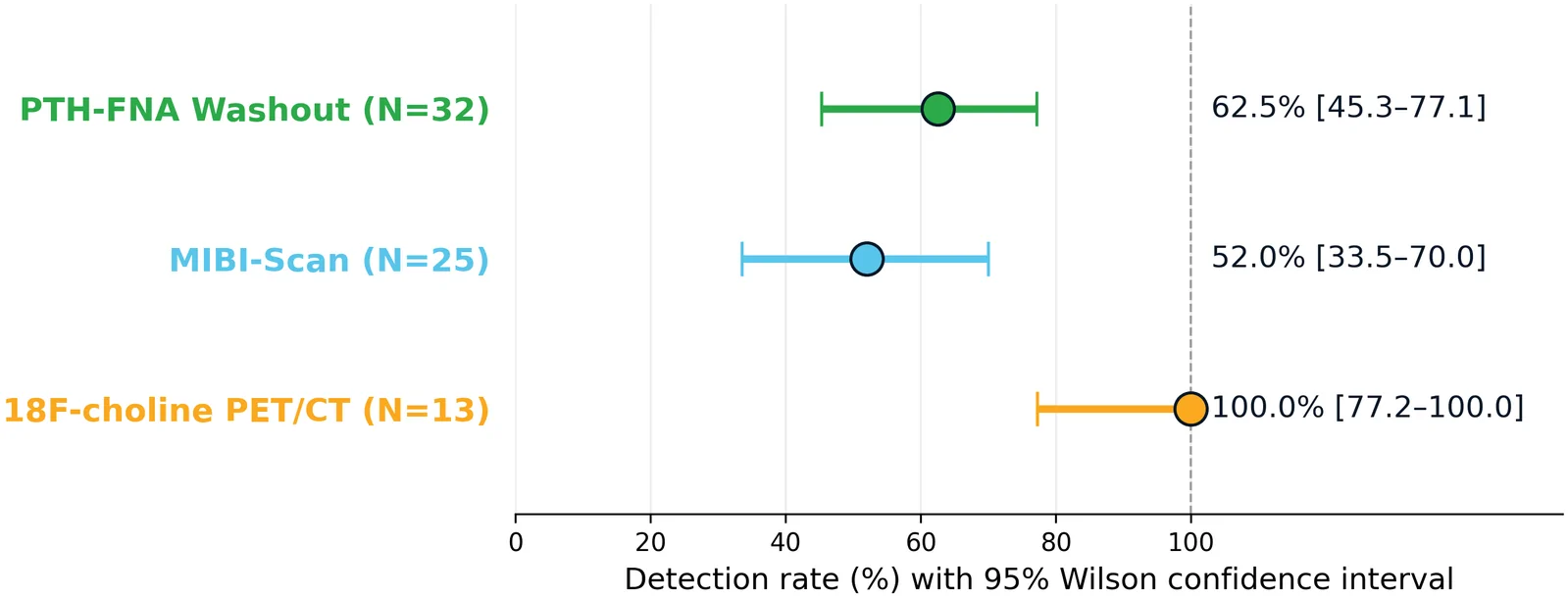
Detection rates by diagnostic modality (PTH-FNA washout: parathyroid hormone assay on fine-needle aspiration washout; MIBI-Scan: ^99^mTc-sestamibi scintigraphy; ^18^PET/CT: 18F-choline positron emission tomography/computed tomography), with 95% confidence intervals (CI, Wilson score method). In this series, every positive result for all three modalities was concordant with the surgical site, so this measure was 100% throughout; detection rate therefore reflects the overall proportion of patients successfully localized by each modality. Because all 32 patients had surgically confirmed single-gland disease, true negatives could not be defined and specificity/NPV are not shown. The reference standard for concordance was surgical and histopathological confirmation of the affected parathyroid gland, as in **Figure 2**. N, number of patients.

^18^F-choline PET/CT was positive for localization in 13/13 (100%, 95% CI 77.2–100%) cases (**Figure 3**). Every positive result was concordant with the confirmed surgical site for all three modalities: PTH-FNA washout (20/20 positive results, 100%, 95% CI 83.9–100%), MIBI-Scan (13/13, 100%, 95% CI 77.2–100%), and ^18^F-choline PET/CT (13/13, 100%, 95% CI 77.2–100%); consequently, concordance with the localized surgical lesion among positive results was 100% for all three tests in this series. Because all 32 patients had surgically confirmed single-gland disease, true negatives could not be defined, and specificity/NPV are not estimable in this design; we therefore report detection and concordance rates rather than sensitivity/specificity throughout. The absence of any site-discordant positive result in this cohort should be interpreted cautiously given the small sample size, particularly for MIBI-Scan and ^18^F-choline PET/CT (n = 13 each), for which the 95% CI lower bound (77.2%) indicates that true concordance as low as approximately 77% remains statistically compatible with the observed data.

## Discussion

Preoperative localization in patients with PHPT is particularly important for therapeutic outcomes. Current clinical practice involves US and MIBI-Scan as first-line investigations. US permits the anatomical detection of an enlarged parathyroid gland relative to the thyroid, although co-existing nodular thyroid disease reduces the method’s diagnostic performance (21). The MIBI-Scan is often burdened by a high number of false negatives (7). In our series, it was diagnostic in only 52% (95% CI 33.5–70%) of cases. In clinical practice, many parathyroid lesions missed by the MIBI-Scan are subsequently identified by ^18^F-choline PET/CT (22). In our study population, ^18^F-choline PET/CT confirmed its strong diagnostic power (detection rate: 100%, 95% CI 77.2–100%). However, it often implies that many patients undergo two nuclear medicine diagnostic procedures during their PHPT preoperative work-up, raising the broader question of whether ^18^F-choline PET/CT might eventually replace conventional first-line imaging altogether — a possibility explored by Giovanella et al. (23). Another important localization method is 4D-CT, which enables the detection of hyperfunctioning parathyroid glands even in the proximity of large nodular goiters, a situation in which it is much more difficult to perform a PTH-FNA washout. However, the radiation exposure, high costs, and limited availability at few referral centers severely limit its use to very specific cases; moreover, specific research on this topic is yet to come (10).

In this scenario, the PTH-FNA washout becomes a valuable additional diagnostic method to assess the parathyroid nature of suspicious lesions detected by neck ultrasound. In this cohort, PTH-FNA washout appeared to be a useful adjunctive localization tool in patients with ultrasound-detectable lesions. In our series, this method proved diagnostic/positive in 62.5% (95% CI 45.3–77.1%) of patients. Conversely, although within a biased sampling framework, among the 37.5% of patients where the washout was non-diagnostic, only half were positive on the MIBI-Scan, leading to the use of ^18^F-choline PET/CT as a confirmatory test in 50% of these cases.

Moise et al. previously demonstrated in a population of 87 patients that PTH-FNA washout alone could identify adenomas in 74.7% of patients, while MIBI-Scan confirmed this in only 90.8% of cases (19). It is likely that the difference in localization rate of the PTH-FNA washout test is due not only to the case mix but also to the diagnostic cutoff used. Moise et al., in fact, defined the PTH-FNA washout test as positive when the values were both above 400 pmol/L and at least three times higher than serum levels. Because the washout-to-serum PTH ratio threshold >1 used to define a positive result is markedly more permissive than criteria used in prior series, we performed a *post-hoc* sensitivity analysis using stricter thresholds. Applying a ratio ≥3 reduced the positivity rate from 62.5% (20/32) to 53.1% (17/32), while applying the considerably stricter criterion proposed by Moise et al. (washout level >400 pmol/L ≈3770 pg/mL using the conversion factor above and ≥3-fold the serum level) reduced the positivity rate to 3.1% (1/32). This wide range illustrates that the reported detection rate is highly sensitive to the chosen threshold, and that our permissive cutoff likely represents an upper-bound, best-case estimate of washout performance; it is also clear that the closer the value is to 1, the greater the risk of false positives.

In our descriptive study, 7/32 (21.9%) patients were referred for parathyroidectomy based solely on a positive PTH-FNA washout; in all of these cases, postoperative histopathology confirmed the correct preoperative localization. Furthermore, 6/32 (18.7%) patients referred for surgery had a positive PTH-FNA washout but a negative MIBI-Scan. In 2 of these 6 cases, postoperative examination directly confirmed the washout localization. In the remaining 4 cases, a subsequent ^18^F-choline PET/CT scan was consistent with the washout findings, and postoperative examination again confirmed the localization. By utilizing PTH-FNA washout as a first-line investigation—reserving ^18^F-choline PET/CT as a second-line option for patients with negative washouts—25 patients (78.1%, 95% CI 61.2–89.0%) in our series could potentially have been spared a MIBI-Scan, which ultimately proved negative in 48% of them anyway.

Our study has some limitations. The retrospective design allows us to assess the localization rate in a relatively small cohort of patients selected by the sonographer, rather than in all patients who underwent surgery: this confirms that the study cohort is characterized by easily visualized and accessible adenomas — the most favorable scenario for both ultrasound and flushing during FNA — and that the detection rates reported should not be extrapolated to patients with lesions that are unlocalizable or technically inaccessible. A second, related limitation is verification (work-up) bias: the order and choice of subsequent imaging were not randomized but were determined by the result of the preceding test—patients with a positive washout more often proceeded directly to surgery or bypassed MIBI-Scan, whereas those with a negative or equivocal result were preferentially escalated to MIBI-Scan and, if still non-localizing, to ^18^F-choline PET/CT. As a consequence, MIBI-Scan (n = 25) and ^18^F-choline PET/CT (n = 13) were performed in non-random, outcome-dependent subgroups rather than in the full cohort, and their reported yields are therefore not directly comparable to one another or to the washout yield, as each is calculated on a different, selectively enriched denominator; the paired McNemar comparison reported above, restricted to patients who underwent both washout and MIBI-Scan, partially mitigates this issue but cannot eliminate it. Compounding this issue, MIBI-Scan and ^18^F-choline PET/CT were interpreted by the reporting physicians as part of routine clinical care, without blinding to the prior ultrasonography or PTH-FNA washout results; unblinded interpretation in a sequential work-up can introduce reviewer bias, particularly for equivocal images, and represents an additional source of potential overestimation of concordance between modalities. Prospective observational studies are needed to validate the diagnostic performance of the PTH-FNA washout against the aforementioned methods.

In addition, the cutoff used to determine a positive washout was arbitrary, as there is no universally validated cutoff in the literature. Some authors consider any FNA-PTH positive that is above the serum PTH level, whereas others suggest an FNA-PTH/serum PTH ratio of ≥ 2:1 in order to minimize the influence of sample contamination by blood (14). Maser et al. reported that a PTH-FNA washout value above the normal serum range (6–40 pg/mL) indicated parathyroid tissue, while 49–65 pg/mL was considered suspicious (20). Based on our data, we believe a negative PTH-FNA washout generally reflects a ratio of washout PTH to serum PTH of < 1. Another limitation of this study is the operator-dependent nature of both neck ultrasonography and PTH-FNA washout, as variations in technical expertise may have influenced lesion detection, washout accuracy, and the overall detection rate observed in our cohort. PTH-FNA washout can only be performed on lesions visible on ultrasound and located in accessible areas. Hyperplastic parathyroids are sometimes found in atypical positions (e.g., the anterior mediastinum) and can only be located via MIBI-Scan and/or ^18^F-choline PET/CT (24).

Let it be noted that our work supports the role of the PTH-FNA washout as a simple and safe first-line investigation for preoperatively localizing hyperplastic parathyroids, as part of a stepwise diagnostic proposal rather than a validated pathway. These findings suggest that the role of MIBI-Scan in this setting may warrant reassessment, while ^18^F-choline PET/CT appears to be a valuable confirmatory modality in non-localizing cases. Naturally, the MIBI-Scan retains its value as a first-line investigation when US is negative or when lesions are not amenable to FNA. This stepwise approach may reduce the use of additional imaging, although formal cost-effectiveness analyses are needed. A brief consideration of the cost/radiation trade-off supports this rationale: PTH-FNA washout itself carries no radiation exposure, whereas MIBI-Scan delivers an estimated effective dose of approximately 6.8–7.4 mSv and dual-phase ^18^F-choline PET/CT approximately 2.8 mSv (25); reserving MIBI-Scan for washout-negative or FNA-inaccessible lesions, as proposed here, would in principle reduce cumulative radiation exposure and resource use for the subset of patients successfully localized by washout and ultrasonography alone, though this potential benefit remains to be formally quantified. Four-dimensional CT (4D-CT), another second-line localization option not used in our institution, offers high spatial resolution but at a substantially higher radiation dose (approximately 5.6–28 mSv) than either MIBI-Scan or ^18^F-choline PET/CT (26), reinforcing ^18^F-choline PET/CT as an attractive confirmatory option where available. Current imaging-specific guidelines do not mandate a single preoperative localization algorithm, leaving room for institution-level strategies such as the one proposed here (26, 27); further prospective, multicenter studies will be needed before PTH-FNA washout can be recommended as a formal component of consensus localization algorithms.

## Conclusion

Our study, grounded in real-world clinical experience, is consistent with the use of PTH-FNA washout in the preoperative diagnostic work-up of primary hyperparathyroidism, particularly where ultrasound expertise is available. Its safety profile is high, though performance depends on operator experience. In this retrospective single-center study, PTH-FNA washout combined with ultrasonography was a useful approach for preoperative localization in selected patients with primary hyperparathyroidism and ultrasound-detectable lesions. We propose a stepwise strategy incorporating ^18^F-choline PET/CT in non-localizing cases as a means to potentially optimize the use of conventional nuclear imaging; this proposal, rather than a validated diagnostic pathway, requires confirmation in larger prospective studies before adoption into routine practice.

## Data Availability

The raw data supporting the conclusions of this article will be made available by the authors, without undue reservation.
